# Metabolomics Analysis Revealed the Characteristic Metabolites of Hemp Seeds Varieties and Metabolites Responsible for Antioxidant Properties

**DOI:** 10.3389/fpls.2022.904163

**Published:** 2022-06-21

**Authors:** Kang Ning, Cong Hou, Xiuye Wei, Yuxin Zhou, Shuanghua Zhang, Yongzhong Chen, Haibin Yu, Linlin Dong, Shilin Chen

**Affiliations:** ^1^Key Laboratory of Beijing for Identification and Safety Evaluation of Chinese Medicine, Institute of Chinese Materia Medica, China Academy of Chinese Medical Sciences, Beijing, China; ^2^Yunnan Hemp Industrial Investment CO.LTD, Kunming, China

**Keywords:** hemp seed, WGCNA, flavone, fatty acid, antioxidant activity

## Abstract

Hemp seeds are rich in metabolites such as protein, lipids and flavonoids, which are beneficial to health and can be used as a nutritional supplement. Few studies have focused on the metabolites of different hemp seed varieties. In the current study, using widely targeted metabolomics based on UHPLC-QQQ-MS/MS, we compared the metabolomes of seeds from seven hemp varieties with different uses. A total of 1,001 metabolites, including 201 flavonoids, 86 alkaloids, and 149 phenolic acids, were identified. Flavonoids, organic acids, alkaloids, lipids, and fatty acids with high nutritional value are important to investigate the differences between hemp accessions. By using weighted gene co-expression network analysis (WGCNA), six modules of closely related metabolites were identified. And, we identified the metabolite characteristics and hub metabolites of each variety. Then, we experimentally determined antioxidant activity of seven varieties and demonstrated that alkaloids, flavonoids, phenolic acids, terpenes, and free fatty acids are responsible for the antioxidant activity of hemp seeds. Our research provides useful information for further investigation of the chemical composition of hemp seeds.

## Introduction

Hemp (*Cannabis sativa* L.), an annual plant belonging to the *Cannabaceae* family, has been cultivated for years (Andre et al., 2016). Morphological variability and versatility as well as the biochemical diversity make this plant a multipurpose plant used for nutrients (seed), fuel (seed), textile (stalks), and medicine (seed and inflorescence). Hemp is also used for fodder, construction, and cosmetics (Hurgobin et al., 2021). Hemp, a controversial plant, is restricted by law, mainly because it contains a variety of cannabinoids including Δ9-tetrahydrocannabinol (THC) and cannabidiol (CBD). According to the content of Δ9-THC, hemp can be classified into two types, namely, drug type (THC > 0.3%) and non-drug type (THC < 0.3%; Kovalchuk et al., 2020). Hemp is a crop of multifunctional usage, and the non-drug type can be further divided into use as food and fodder, fiber use, and medicinal use (Schluttenhofer and Yuan, 2017).

Hemp seeds are a rich source of high-quality proteins and oils, and have been an important source of nutrition since ancient China. Hemp seeds contains over 30% protein, 25% starch, and 30% oil, and considerable amounts of dietary fiber and minerals (House et al., 2010; Schluttenhofer and Yuan, 2017). Hemp seeds were known to be an excellent source of digestible protein, which is available for absorption due to the large proportion of seed storage protein (Aiello et al., 2016). Edestin, a type of hexameric legumin, is easily digested and contains nutritionally significant amounts of all essential amino acids (Park et al., 2012). Moreover, hemp seed protein also has potential applications as nutraceuticals. Two hemp seed proteomic studies enriched the future study of protein in hemp seeds (Park et al., 2012; Aiello et al., 2016). Hemp seed also contains a large amount of polyunsaturated fatty acids (PUFAs), including linoleic and α-linolenic acids (approximately 53% and 18% of total fatty acids, respectively) and a small amount of other fatty acids that are not commonly found in vegetable oils, such as γ-linolenic acid and stearidonic acid (Simopoulos, 2008). Hemp seed oil contains over 90% polyunsaturated fatty acids, making it a valuable addition to human and animal diets (Gomez et al., 2011). Hemp seeds also contain many metabolites, such as unsaturated fatty acids, flavone, natural phenols, cannabinoids, and tocopherols that contribute to the antioxidant activity (Chen et al., 2012; Rashid et al., 2021). Hemp seeds and hemp seed oil are believed to have a positive effect on the human health. For example, hemp seed and hemp seed oil benefit the cardiovascular system in genetically obese individuals by improving the cell-mediated immune response (Abrahamsen et al., 2021; Majewski and Jurgoński, 2021). Hemp seeds are also a traditional Chinese medicine believed to be beneficial for intestinal relaxation.

Selection of malic acid and triterpene metabolites during domestication leads to edible properties of Jujube (Zhang et al., 2021), suggesting that genomic selection caused by domestication and/or environment can lead to markedly different varieties. Hemp originated in Central Asia and gradually spread around the world and domesticated into varieties with different characteristics. During the domestication process, varieties of hemp with different properties have been produced for different uses. For example, loss of function of two cannabinoid synthases has occurred during selection of fiber-types and medicinal-types (Ren et al., 2021). Previous study on more than 20 hemp breeding varieties revealed significant differences in antinutritional components, oil content, and protein content (Galasso et al., 2016), suggesting a great influence of domestication on variation in hemp. Reproduction conditions, such as agronomic and climatic conditions, can also affect metabolites in hemp seeds. Metabolome analysis showed that 236 metabolites were differentially accumulated in two local hemp seed breeding varieties with different dimensions in India (Rashid et al., 2021). Being planted in the mountain environment greatly affects the fatty acids profile and carotenoids content of hemp seeds (Cattaneo et al., 2021). Proteomic signatures also indicated that there are differences between two hemp seed varieties (Park et al., 2012; Aiello et al., 2016). As a multifaceted crop, the target trait of breeding is highly correlated with the usage of the variety. For example, the plant with high content of CBD can make great contribution for medical use variety. Plant with high content protein and oil seeds might be ideal parents for food/fodder use variety (Schluttenhofer and Yuan, 2017). As a plant organ with important nutritional and medical value, few studies have focused on the metabolite variation in different hemp seed varieties, resulted in a shortage of information of new hemp varieties with ideal seeds traits. Widely targeted metabolomics has been applied in plant metabolite analysis in many species due to the advantages of high-throughput and high sensitivity. So, it can be an effective approach in performing qualitative and quantitative analyses to determine the nutrient composition of hemp seeds. In this study, we used widely targeted metabolomics to explore the types and distribution of metabolites in seven hemp seeds varieties with significant phenotypic differences. Further, the antioxidant and radical scavenging studies revealed that terpenes and phenols attribute the antioxidant activity in hemp seeds. Our study provides useful information for further improvement of hemp seeds.

## Materials and Methods

### Plant Materials, Sample Preparation, and Metabolite Extraction

**Figure 1:**
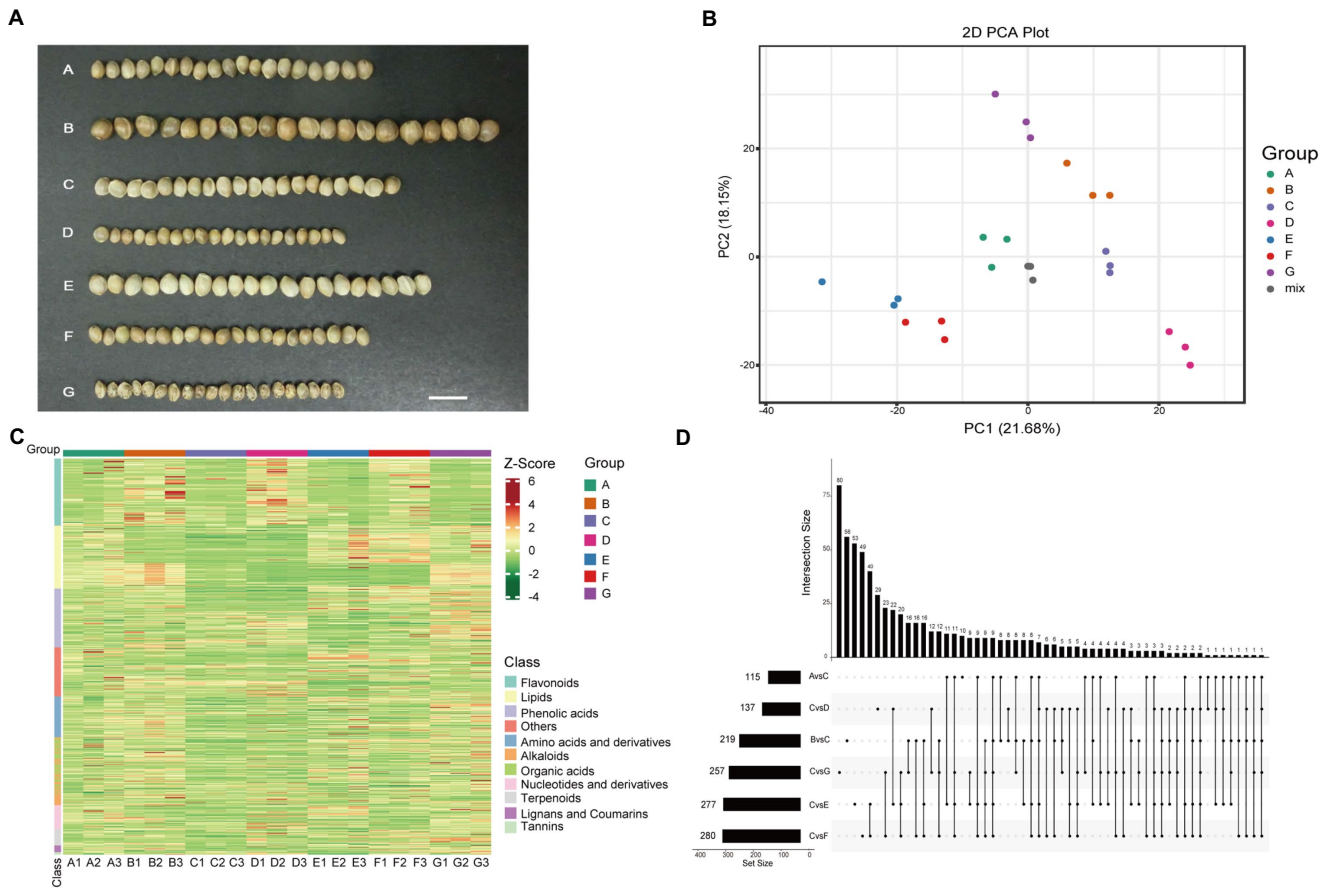
Overview of the detected metabolites in the seven hemp seed varieties. **(A)** Photographs of seven hemp seed varieties. **(B)** PCA score plot. **(C)** Clustering heatmap of all metabolites. Each sample is represented by a column, and each metabolite is represented by a row. The abundance of each metabolite is represented by a bar with specific color. The upregulated and downregulated metabolites are indicated by different shades of red and green, respectively. **(D)** Number of metabolites that detected in different comparison.

The seeds of seven local breeding varieties were collected in the growing season of 2020. Seeds were harvested when 80% brown seed appeared and stored at 5°C. Seeds from seven varieties (A–G) with significant difference in phenotype and origin were selected for the present study (Figure 1A; Table 1; Supplementary Table 1). The B variety has the largest size and weight among all varieties, while the D and G varieties are the smallest in size among all varieties. The B variety has the darkest seed coat color, while the E variety has the lightest seed coat color. And, the G variety has black spots on the seed coat. Plants of A, C, and G varieties contain high level of cannabinoids and they are used for the CBD extraction. While, plants of B, D, E, and F varieties have low level of cannabinoids and they are food-use type or fiber-use type.

**Table 1:** Characteristic of seven hemp seed varieties.

Variety	Width	Length	Weight	Seed coat color	Type
A	4.08	5.81	332.7125	Gray	Medicinal use
B	5.16	6.83	737.325	Yellow	Fiber/food use
C	4.01	5.87	360.5375	White	Medicinal use
D	3.22	4.84	196.1375	Dark	Fiber/food use
E	4.51	6.42	400.25	White	Fiber/food use
F	3.71	5.55	234.5	Dark	Fiber/food use
G	3.14	4.62	178.1786	Stripe	Medicinal use

Whole seeds (un-dehulled) of seven varieties with three biological replicates were subjected to analysis by widely targeted metabolomics (Metware Biotechnology Co., Ltd. Wuhan, China). Samples were freeze-dried by a vacuum freeze-dryer (Scientz-100F) and then crushed using a mixer mill (MM 400, Retsch, Haan, Germany) with a zirconia bead for 1.5 min at 30 Hz. Samples were dissolved (100 mg of lyophilized powder) in 1.2 ml of 70% methanol solution, vortexed for 30 s every 30 min (total of six times), and then stored at 4°C overnight. Following centrifugation at 12,000 rpm for 10 min, the extracts were filtrated (SCAA-104, 0.22 μm pore size; ANPEL, Shanghai, China) before UPLC-MS/MS analysis. Quality control (QC) samples were prepared by mixing sample extracts. During analysis, a QC sample was included in the measurement queue every six test samples to monitor measurement repeatability, i.e., one QC before the first sample, one QC after the first six samples, one QC after the second six samples, and one QC after the last six samples.

### UPLC-ESI-Q TRAP-MS/MS

The sample extracts were analyzed using an UPLC-ESI-MS/MS system (UPLC, SHIMADZU Nexera X2, Shanghai, China; MS, Applied Biosystems 4,500 Q TRAP, Thermo Fisher, Shanghai, China) with an Agilent SB-C18 column (1.8 μm, 2.1 mm × 100 mm). The mobile phase consisted of solvent A (pure water with 0.1% formic acid) and solvent B (acetonitrile with 0.1% formic acid). Sample measurements were performed with the following gradient program: starting conditions of 95% A and 5% B; a linear gradient to 5% A and 95% B was adjusted within 9 min and kept for 1 min; and a composition of 95% A and 5.0% B was adjusted within 1.1 min and kept for 2.9 min. The flow velocity was set as 0.35 ml per min, and the column oven was set to 40°C. The injection volume was 4 μl. The effluent was alternatively connected to an ESI-triple quadrupole-linear ion trap (QTRAP)-MS.

### ESI-Q TRAP-MS/MS

LIT and triple quadrupole (QQQ) scans were acquired on a triple quadrupole-linear ion trap mass spectrometer (Q TRAP; AB4500 Q TRAP UPLC/MS/MS System, Milwaukee, United States) equipped with an ESI Turbo Ion-Spray interface, operating in positive and negative ion mode, and controlled by Analyst 1.6.3 software (AB Sciex). The ESI source operation parameters were as follows: ion source, turbo spray; source temperature, 550°C; ion spray voltage (IS), 5,500 V (positive ion mode)/−4,500 V (negative ion mode); ion source, gas I (GSI), gas II (GSII), and curtain gas (CUR) set at 50, 60, and 25.0 psi, respectively; and collision-activated dissociation (CAD), high. Instrument tuning and mass calibration were performed with 10 and 100 μmol/L polypropylene glycol solutions in QQQ and LIT modes, respectively. QQQ scans were acquired as multiple reaction monitoring (MRM) experiments with collision gas (nitrogen) set to medium. Declustering potential (DP) and collision energy (CE) for individual MRM transitions were performed with further DP and CE optimization. A specific set of MRM transitions were monitored for each period according to the metabolites eluted within this period.

### Qualitative and Quantitative Analysis of Metabolites

Based on the self-built database MWDB V2.0 (Metware Biotechnology Co., Ltd. Wuhan, China), primary and secondary mass spectrometry data were subjected to qualitative analysis. Isotopic signals, repeating signals containing K^+^ ions, Na^+^ ions, NH4^+^ ions, and repeating signals of fragment ions that are themselves other larger molecular weight species were removed from the analysis.

Metabolite quantification was performed using triple-quadrupole mass spectrometry in multiple reaction monitoring (MRM). In MRM mode, the quadrupole first screens the precursor ions (parent ions) of the target substance, and excludes the ions corresponding to other molecular weight substances to preliminarily eliminate interference; precursor ions are induced to ionize by the collision cell and then fragmented to form many fragment ions. The fragment ions are then filtered through triple quadrupole to select a desired characteristic fragment ion, eliminating the interference of non-target ions, making the quantification more accurate and repeatable. After obtaining the metabolite spectrum analysis data of different samples, perform peak area integration on all the mass spectrum peaks of the substances, and perform integration correction on the mass spectral peaks of the same metabolite in different samples (Fraga et al., 2010). In order to compare the differences in the content of each metabolite in different samples among all the detected metabolites, we corrected the mass spectral peaks detected in different samples for each metabolite according to the information of metabolite retention time and peak shape, in order to ensure the accuracy of qualitative and quantitative. The relative contents of metabolite in each sample were represented with chromatographic peak area integrals.

### Multivariate Data Analysis and Statistical Analysis

Data were normalized with sum, log transformation, and auto-scaling before any statistical analysis. Unsupervised principal component analysis (PCA) was performed by the “prcomp” statistics function within R.^1^ The data were unit variance scaled before unsupervised PCA. The hierarchical cluster analysis (HCA) results of samples and metabolites are presented as heatmaps with dendrograms, while Pearson correlation coefficients (PCC) between samples were calculated by the “cor” function in R and are presented as heatmaps. Both HCA and PCC were performed using the “pheatmap” package in R. For HCA, the normalized signal intensities of metabolites (unit variance scaling) are presented as a color spectrum. Significantly different accumulated metabolites (DAMs) between groups were determined by VIP (Variable importance in the projection) ≥ 1 and absolute log_2_ (fold change) ≥ 1. VIP values were extracted from the OPLS-DA (orthogonal partial least squares discriminant analysis) results, which also contained score plots and permutation plots, and they were generated using the “MetaboAnalystR” package in R. The data were log transformed (log2) and mean centered before OPLS-DA. To avoid overfitting, a permutation test (200 permutations) was performed. Student’s *t*-test was carried out using IBM SPSS Statistics 25 Software to identify the significant differences at α level of 0.05 for all biochemical analyses.

### WGCNA and Metabolite Network Visualization

The metabolites that detected in at least two replicates of any sample were used for WGCNA (version: WGCNA_1.70-3). The co-expression modules were obtained by using one-step network construction function with following parameters: soft threshold power: 20, TOMtype: unsigned, mergeCutHeight: 0.25 and minModuleSize: 30, other parameters: default. The initial clusters were merged on eigen-metabolites. Eigen-metabolites were calculated for each module, which were used to search the association with metabolite characteristics of varieties (Zhang and Horvath, 2005). The analysis results of WGCNA were exported to the cytoscape software (v3.7.0, United States) to generate metabolite interaction networks. Edges with weight less than 0.2 are removed due to excessive number of edges. The importance of metabolites was ranked based on the degree of metabolites to select the hub metabolites of modules.

### Kyoto Encyclopedia of Genes and Genomes (KEGG) Annotation and Enrichment Analysis

Identified metabolites were annotated using the KEGG compound database,^2^ and annotated metabolites were then mapped to the KEGG Pathway database.^3^ Pathways with significantly regulated metabolites were then subjected to metabolite set enrichment analysis (MSEA), and their significance was determined by hypergeometric test *p*-values.

### Determination of Total Antioxidant Capacity and Antioxidant Analysis

Quantification of antioxidant activities was performed on a dry weight basis. Liquid nitrogen was added to the seven samples, which were then ground to pass a 60 mesh-sieve with an IKA all basic mill (IKA Works Inc., Wilmington, NC, USA). The powder was then freeze-dried to constant weight. A portion of the powder (0.2 g) was extracted in a capped centrifuge tube with 4 ml of solvents including 80% ethanol and 20% distilled water. The mixture was ultrasonically extracted at 50°C for 20 min at 38 kHz and 30 kW. The extracts were then centrifuged by an Allegra 21R Centrifuge (Beckman Coulter Ltd., Palo Alto, CA, USA) at 4500 rpm for 10 min, and the supernatants were removed into new tubes. Residues were extracted with the same solvents for the second time. Both extracts were combined and stored at 4°C in the dark until further analysis within 2 d. Extractions were performed in three replicates for each sample.

### 1,1-Diphenyl-2-Picrylhydrazyl Radical-Scavenging Activity

The 1,1-diphenyl-2-picrylhydrazyl (DPPH) radical-scavenging capacity of hemp seed extracts was evaluated according to the method of (Xie et al., 1993) with slight modifications. Briefly, 1 ml of the tested hemp seed extract was added to 3 ml of methanol solution containing DPPH radicals (final concentration of 0.1 mM). The mixture was shaken vigorously for 1 min by vortexing and left to stand at room temperature in the dark for 30 min. Thereafter, the absorbance of the sample (A_sample_) was measured using an UV 160 spectrophotometer at 517 nm against an ethanol blank. The absorbance of a complete blank control (A_c-blank_) was measured after adding 3 ml of methanol to 1 ml of ethanol. The absorbance of a blank control (A_blank_) was measured after adding 3 ml of methanol to 1 ml of the sample solution. The absorbance of a negative control (A_control_) was measured after adding DPPH solution to 1 ml of the extraction solvent. The percent of DPPH discoloration of the sample was calculated according to the following equation:

$\begin{array}{l} \mathrm{Percent discoloration}\\ =\{1-[\left(A_{\mathrm{sample}}-A_{\mathrm{blank}}\right)/(A_{\mathrm{control}}-A_{c-\mathrm{blank}}\}\times 100 \end{array}$ .

### Ferric Reducing Antioxidant Power Assay

The ferric reducing antioxidant power (FRAP) assay was performed as previously described by (Benzie and Strain, 1996) with some modifications. The sample solution analyzed was first diluted with deionized water to fit within the linearity range. The test was performed according to the manufacturer’s instructions (WARYONG, Beijing, China). The working FRAP reagent was prepared by mixing 1 ml of distilled water with 20 μl of 98% H_2_SO_4_ and 10 mg of FeSO_4_·7H_2_O. Then, 900 μl of working FRAP reagent was warmed to 37°C, and 30 μl of sample and 90 μl of deionized water were added to the FRAP reagent. The absorbance was then measured at 593 nm against a reagent blank after 10 min. The FRAP value was calculated and expressed as micromoles of Fe^2+^ equivalent (FE) per gram of hemp seed sample using the calibration curve of Fe^2+^. The linearity range of the calibration curve was 0.003125 to 0.1 μmol (r = 0.9996).

## Results and Discussion

### Metabolomics Profiling of Different Hemp Seed Varieties

Widely targeted metabolomics was used to explore the differences in metabolites among the hemp seed varieties. Based on the comparison of identified mass spectra with the local metabolite database, a total of 1,001 metabolites have been identified, including 43 terpenoids, six tannins, 149 phenolic acids, 87 organic acids, 61 nucleotides and derivatives, 159 lipids, 18 lignans and coumarins, six chalcones, 13 flavanones, five flavanonols, nine anthocyanidins, 51 flavones, 55 flavonols, 14 flavonoid carbonosides, eight flavanols, 12 isoflavones, 103 amino acid and derivatives, 86 alkaloids, 47 saccharides, 18 alcohols, 13 vitamins, 10 cannabinoids, and 28 others metabolites. The detailed information of these metabolites was shown in Supplementary Table 2. Previous studies have shown that hemp seeds contain a high quantity of essential amino acids and oil. In total, we identified 103 amino acids and derivatives, including essential amino acids, as well as 159 lipids, including free fatty acids, LPCs and LPEs, in the metabolome of hemp seed, indicating that hemp seeds are ideal for nutrition. Polyunsaturated fatty acids, such as linolenic acid, were also highly accumulated in hemp seeds. Moreover, secondary metabolites, including 173 flavonoids, 86 alkaloids, and 43 terpenoids, were also detected in hemp seeds, and these secondary metabolites may be beneficial for health. Eighteen lignans and coumarins may constitute the seed coat (Yonekura-Sakakibara et al., 2021). Cannabinoids are mainly produced in female inflorescences, especially in glandular trichomes located on bracts (Andre et al., 2016; Kovalchuk et al., 2020). Ten cannabinoids were also detected in metabolome, but they were irregularly distributed among varieties and replicates, suggesting that the detected cannabinoids were not synthesized in the seed but a residue from the glandular trichomes.

PCA showed that the different varieties were separated with PC1 and PC2 accounting for 21.68% and 18.15%, respectively (Figure 1B). High correlation coefficients (*r* = 0.915–0.991) were observed among the three biological replicates of each sample (Supplementary Figure 1A), suggesting that the data are reproducible and reliable. There were 973, 964, 939, 968, 947, 990, and 944 metabolites detected in the A, B, C, D, E, F, and G varieties, respectively. Eight hundred and fifty-four metabolites were commonly detected in all seven varieties (Supplementary Figure 1B). The proportions of each class of metabolites were similar in all varieties. Of these, lipids, flavonoids, and phenolic acids were the major metabolites (Supplementary Figure 1C). The heatmap showed that the flavonoids were highly abundant in the B, D, and F varieties, and the A and G varieties were rich in lipids compared to the other varieties. Meanwhile, the G variety was rich in tannins, lignans, and coumarins (Figure 1C). These results indicated that the difference between the presented hemp varieties lies in the representation of individual components in the metabolomic profiles of the samples, while the qualitative composition is approximately the same.

### Identification of Metabolites Responsible for Differences Among Seven Varieties

The difference of phenotypes among the hemp seed varieties is mainly in the contents of some metabolites. We use the supervised method, OPLS-DA and Student’s *t* (value of *p*<0.05) test, to find the metabolites responsible for differences among these seven varieties. In this study, the OPLS-DA model compared metabolite contents of the varieties in pairs to evaluate the differences. The Q^2^ values of all comparison groups exceeded 0.8 except A vs. E, demonstrating that the models were stable (Li et al., 2021). The DAMs (|Log (FC)| > 1, VIP ≥ 1 and value of *p* < 0.05) between varieties were screened (Supplementary Table 3). Most of the metabolites were not highly accumulated in C variety, therefore, C variety was used to compared with other varieties. The number of DAMs in different comparison groups (C vs. A, B, D, E, F, and G) was shown in Figure 1D. The upset diagram showed that only two metabolites were shared among the comparison groups (C vs. A, B, D, E, F, and G). These results showed that the metabolites that caused the differences between C and A, B, D, E, F, and G were greatly different.

### Identification of Modules of Closely Related Metabolites

**Figure 2:**
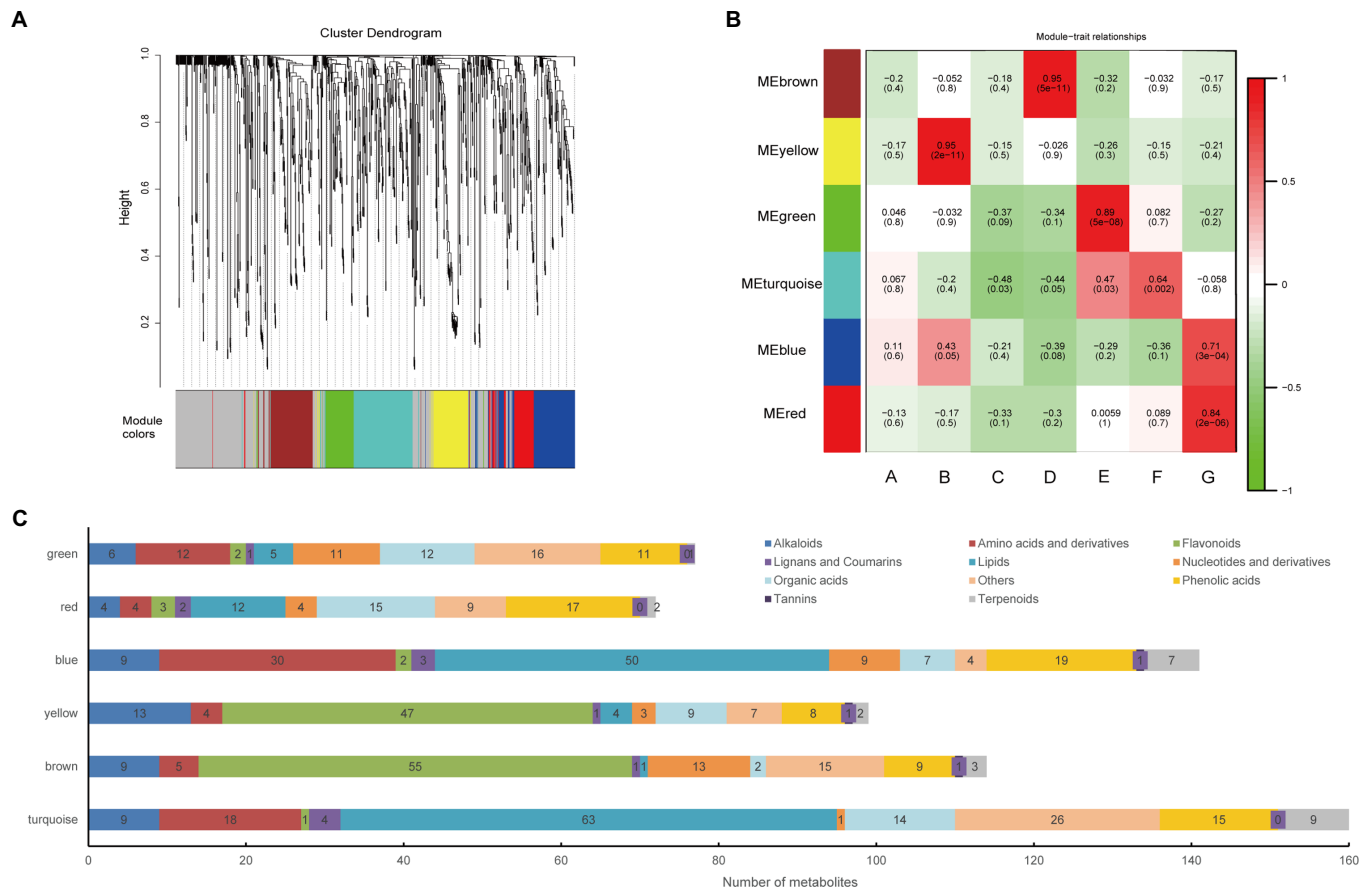
Correlation of metabolites with the hemp seed varieties based on WGCNA. **(A)** Clustering dendrogram of the average network adjacency for the identification of metabolite co-expression modules. Clustering dendrogram of metabolites, with dissimilarity based on topological overlap, together with assigned module colors. **(B)** Module-Variety associations. Each row corresponds to a module eigengene, column to a variety. Each cell contains the corresponding correlation and value of *p*. The table is color-coded by correlation according to the color legend. **(C)** Distribution of different types of metabolites in six modules.

WGCNA is a powerful tool that can identify metabolite sets with highly synergistic changes. Compared with only focusing on differential metabolites, WGCNA uses the information of thousands or nearly tens of thousands of metabolites with the greatest changes or all metabolites to identify the metabolite set of interest and conducts significant association analysis with the phenotype. Thus, we performed WGCNA and constructed a network to further explore the relation between metabolites and hemp seed varieties. The metabolites have been clustered into six modules (comprised of 72–160 metabolites) and those who belong to none of these modules are indicated by grey (Figures 2A,B). Figure 2C shows the composition of the metabolites in the six modules. The details of the metabolites in each module were shown in Supplementary Table 2. The eigen-metabolite, which represented the accumulation profile of the metabolites contained in the module were calculated for each module. Further, we associated each of the co-expression modules with varieties of hemp seed *via* Pearson correlation coefficient analysis (Figure 2B).

### Identification of Metabolite Characteristics in different Varieties

Based on the data of WGCNA, we further identified the hub metabolites of the modules and analyzed the correlation between modules and hemp seed varieties.

**Figure 3:**
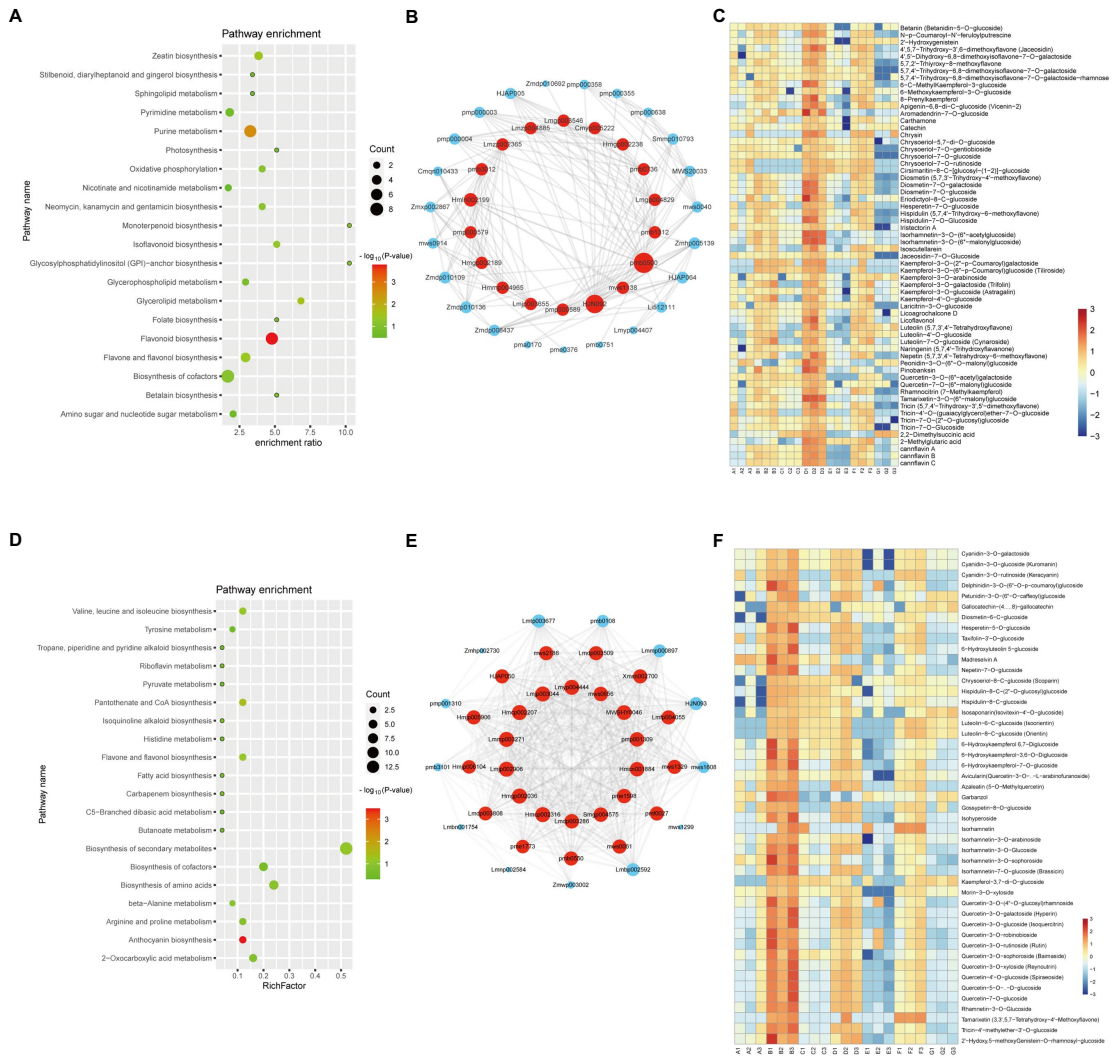
Metabolite characteristics of the D and B varieties. KEGG analysis of the metabolites in the brown **(A)** and yellow **(D)** modules. Each bubble in the plot represents a metabolic pathway whose abscissa and bubble size jointly indicate the magnitude of the impact factors of the pathway. A larger bubble size indicates a larger impact factor. The abscissa of bubbles indicates the enrichment ratio of metabolites in each pathway. The bubble colors represent the value of *p*s of the enrichment analysis, with lighter colors showing a higher confidence level. Sorted by value of *p*, the top 20 metabolic pathways are plotted in the bubble chart. The network of top hub metabolites in red **(B)** and green **(E)** module is indicated by larger circles and the red color in the network. Heatmap showing the accumulation pattern of representative metabolites in brown **(C)** and yellow **(F)** module. Red and bule represent high and low expression, respectively.

The brown module, which contained 114 metabolites, has a high correlation (*r* > 0.8, value of *p* < 0.05) with the D variety (Figure 2B). It indicated that there was a significant association between eigen-metabolite of brown and D variety trait. “Flavonoid biosynthesis,” “flavone and flavonol biosynthesis,” and “isoflavonoid biosynthesis” pathways related metabolites were highly accumulated in D variety (Figure 3A). In the D variety, 55 flavonoids, such as chrysoeriol, kaempferol, and luteolin, as well as their derivatives were detected at a high level. Two alkaloids (p-Coumaroylferuloylputrescine and N-p-Coumaroyl-N′-feruloylputrescine) and derivatives of tricin and diosmetin were hub metabolites in the brown module (Figure 3B). Betanin (mws1138), a natural pigment with powerful antioxidant activity, was also a hub metabolite detected at high levels in the D variety. Cannflavins, which are unique flavonoids in hemp, were also accumulated in the D variety (Figure 3C). The yellow module was highly correlated with the B variety (*r* > 0.8, value of *p*<0.05) and contained 99 metabolites including 47 flavonoids and 13 alkaloids (Figure 2B). Metabolites that involved in “anthocyanin biosynthesis” pathway were highly accumulated in B variety (Figure 3D). Lots of derivatives of quercetin and isorhamnetin are highly accumulated in the B variety and play as hub metabolites in the yellow module (Figure 3E). Meanwhile, several anthocyanins like Cyanidin-3-O-glucoside (Kuromanin) and Cyanidin-3-O-rutinoside (Keracyanin) are clustered into the yellow module (Figure 3F), which might be the reason for the dark color of the seed coat. The diet contains plant flavonoids is thought to have neuroprotective, antioxidant, and anticancer properties (Hostetler et al., 2017; Madunic et al., 2018). B and D varieties have a high level of flavonoids, indicating that both varieties may have high medicinal value.

**Figure 4:**
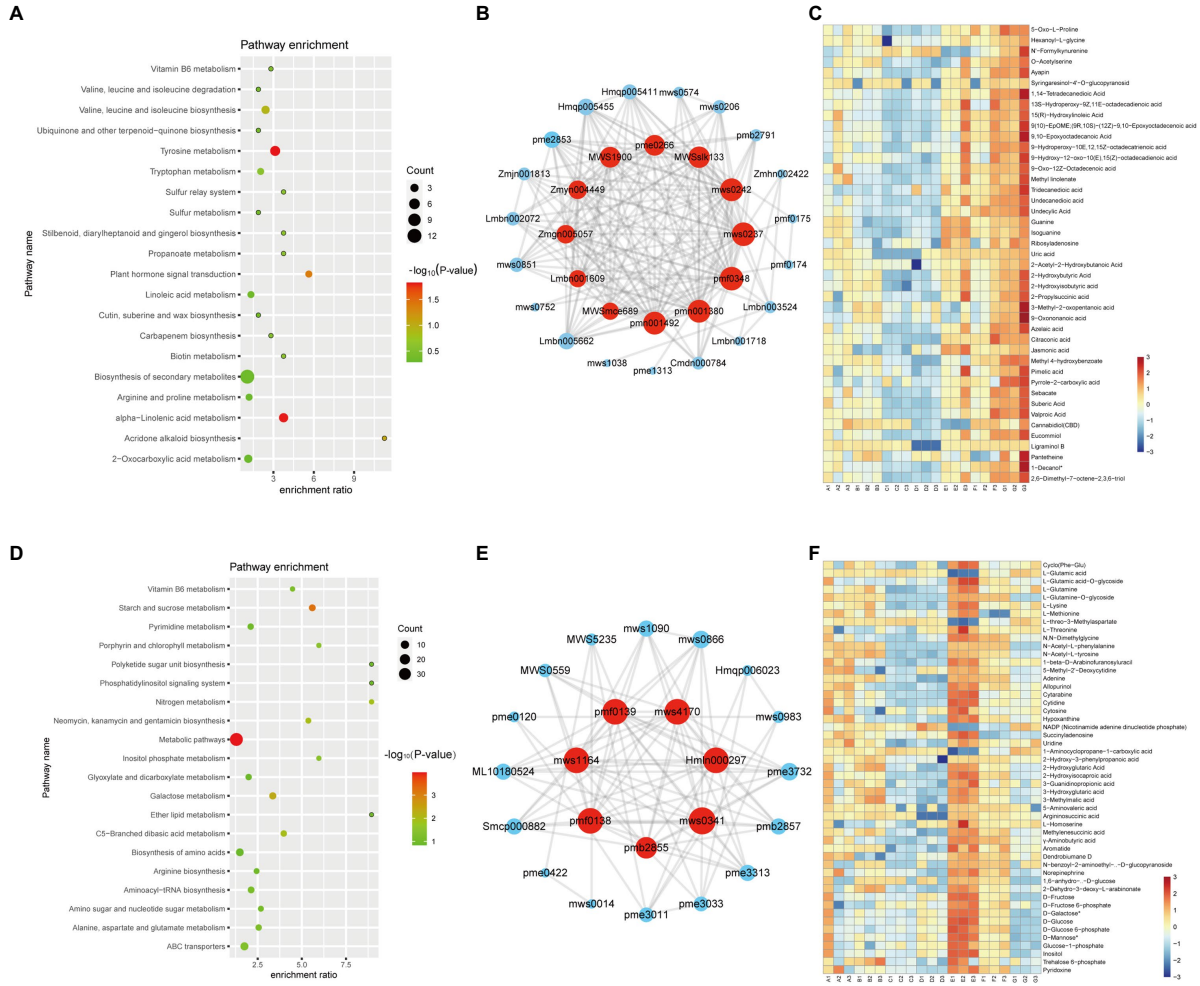
Metabolite characteristics of the E and G varieties. KEGG analysis of the metabolites in the red **(A)** and green **(D)** modules. Each bubble in the plot represents a metabolic pathway whose abscissa and bubble size jointly indicate the magnitude of the impact factors of the pathway. A larger bubble size indicates a larger impact factor. The abscissa of bubbles indicates the enrichment ratio of metabolites in each pathway. The bubble colors represent the value of *p*s of the enrichment analysis, with lighter colors showing a higher confidence level. Sorted by *p*-value, the top 20 metabolic pathways are plotted in the bubble chart. The network of top hub metabolites in red **(B)** and green **(E)** module is indicated by larger circles and the red color in the network. Heatmap showing the accumulation pattern of representative metabolites in red **(C)** and green **(F)** module. Red and bule represent high and low expression, respectively.

The red module contained 72 metabolites and was correlated with the G variety (Figure 2B). Twelve free fatty acids, 15 organic acids, and 17 phenolic acids were clustered in the red module. And, “alpha-linolenic acid metabolism” pathways (value of *p* < 0.05) related metabolites are highly accumulated in G variety (Figure 4A). Several free fatty acids served as hub metabolites in the red module, including methyl-linolenate, undecanedioic acid, and 15(R)-hydroxylinoleic acid (Figures 4B,C). The green module correlated with the E variety, and it was accumulated in the following components: amino acids and derivatives; nucleotides and derivatives; organic acids; and saccharides and alcohols (others class; Figure 2B). “Starch and sucrose metabolism” and “Galactose metabolism” related metabolites are overrepresented in the green module (Figure 4D). Only few secondary metabolites were clustered in the green module. The hub metabolites in the green module were mainly composed of saccharides and alcohols, such as D-Mannose (pmf0138), D-Fructose (mws1164), D-Galactose (pmf0139), D-Glucose (mws4170; Figures 4E,F). E and G varieties are rich in saccharides and fatty acids, suggesting they are ideal varieties for nutrition.

The blue and turquoise modules were overrepresented in different classes of lipids. The lipids clustered in the blue module were lysophosphatidylcholines (LPCs), while the turquoise module contained free fatty acids and glycerol ester. Both modules also contained many amino acids and derivatives (Figure 2C). Although these modules were not significantly correlated with the G and F varieties, these data indicated that these two varieties may be the ideal varieties for oil extraction or consumption.

### Antioxidant Activity and Medicinal Properties Analysis of Hemp Seed

Hemp seeds have been reported to have high antioxidant activity and phenols and flavonoids are thought to contribute to the antioxidant potential in hemp seeds (Frassinetti et al., 2018; Irakli et al., 2019). We investigate the relationship between metabolite profiles of hemp seeds and their antioxidant activities. Firstly, we performed DPPH radical-scavenging and FRAP assays to evaluate the antioxidant activity of the seven varieties (Table 2). The DPPH radical-scavenging activities of the total extract of the hemp seed varieties ranged from 69.71% to 91.91%. The DPPH radical-scavenging activities of the B, D, F, and G varieties were higher than those of the other varieties. The FRAP assay showed that the FRAP of the total extract of hemp seeds ranged from 18.35 μmol Fe^2+^/g to 32.54 μmol Fe^2+^/g. It suggested that there were significant differences in antioxidant activities between hemp seed varieties, and the B, D, F, and G varieties had higher antioxidant activity, while the A and C varieties had lower antioxidant activity.

**Table 2:** Antioxidant activity of seven hemp seed varieties.

Variety	DPPH (%)	TOC values (μmol Fe^2+^/g)
A	70.48 ± 1.41	18.35 ± 0.95
B	88.54 ± 1.45^*^	30.74 ± 0.17
C	69.71 ± 0.57	17.39 ± 1.25^*^
D	88.97 ± 0.70	32.53 ± 0.93^*^
E	80.37 ± 1.65	22.38 ± 1.45
F	86.86 ± 1.82^*^	23.54 ± 0.66^*^
G	91.91 ± 2.44	29.20 ± 0.97

* *Represents statistically significant values at (p* ≤ 0*.05) as determined by Student’s *t*-test.*

Then we preformed WGCNA to calculate the relationship between metabolites and antioxidant activity. Brown, yellow and red modules are highly correlated with antioxidant activity. Several identified metabolites may be responsible for the antioxidant activity of hemp seeds. For example, naringenin and kaempferol-3-O-glucoside, possessing antioxidant, anti-inflammatory, and antiproliferative activities (Li et al., 2021), are found in high levels in hemp seeds. Phenolic acids and alkaloids exhibit strong antioxidant activity such as scavenge free radicals and reduce metal ions (Piazzon et al., 2012; Kumari, 2022). Thus, 2-O-Salicyl-6-O-Galloyl-D-Glucose, Feruloyl syringic acid, 1-O-Feruloyl-D-Glucose (class of phenolic acids) and N-Trans-Feruloyl-3’-O-methyldopamine, N-Feruloyl-3-methoxytyramine, N-Cis-Feruloyl-3’-O-methyldopamine (class of alkaloids) which were highly detected in the B, D, F, and G varieties might attribute the antioxidant activity in hemp seeds. Cannabinoids such as cannabichromenic acid (CBCA), cannabidiolic acid (CBDA), CBD and Δ9-THC also have antioxidant activity (Dawidowicz et al., 2021). Yellow module correlated with B variety was rich in flavonoids such as several derivates of quercetin and isorhamnetin. One phenolic acid and six flavonoids act as hub metabolites in B variety (Figure 3E). Brown module (D variety) contained 55 flavonoids, such as chrysoeriol, kaempferol, and luteolin, as well as their derivatives, and two flavonoids and two alkaloids act as hub metabolites in D variety (Figure 3B). The flavonoid profiles of B and D variety as observed in the metabolome analysis could be the putative reason for their high antioxidant activity. G variety correlated with red module also have high antioxidant activity. And, red module contained many phenols, suggesting that these phenols may also contribute to the antioxidant activity. Most noteworthy, B, D, F, and G varieties with high antioxidant activity have dark coat color. Therefore, anthocyanins like derivates of cyanidin [cyanidin-3-O-galactoside, cyanidin-3-O-glucoside (Kuromanin), and cyanidin-3-O-rutinoside (Keracyanin)], which is responsible for the dark seed coat, are also important metabolites for the antioxidant activity of hemp seeds (Tena et al., 2020). In addition, unsaturated fatty acids also have antioxidant activity. Fatty acids, such as methyl-linolenate, undecanedioic acid, and 15(R)-hydroxylinoleic acid, that highly accumulated in G variety might also contribute to antioxidant activity. The high DPPH radical-scavenging activity in the F variety may be caused by high contents of phenolic acid and terpenoid metabolites. While, the high antioxidant activities of the B and D varieties may be attributed to the flavonoid compounds.

## Conclusion

In this study, by using widely targeted metabolomics, we totally identified 1,001 metabolites in seven different varieties of hemp seeds and found that the content of the metabolites might responsible for the difference of varieties. Then, by using WGCNA, we identified the characteristic metabolites of the hemp seed varieties. Further, antioxidant activity of seven varieties were detected, and we found that flavonoids and phenolics are mainly responsible for the antioxidant activity of hemp seeds. Our research provides helpful information for further investigation of the chemical composition of hemp seeds.

## Data Availability Statement

The original contributions presented in the study are included in the article/Supplementary Material, and further inquiries can be directed to the corresponding authors.

## Author Contributions

KN, CH, and LD designed the research. KN, CH, XW, YZ, YC, and SZ performed the research. KN and CH analyzed the data and wrote the paper. HY, LD, and SC revised the paper. All authors contributed to the article and approved the submitted version.

## Funding

This study was supported by the Fundamental Research Funds for the Central Public Welfare Research Institutes (ZZ13-YQ-049), the Scientific Research Project of Hainan Academician Innovation Platform (SQ2021PTZ0052), and the National Key R&D Program of China from the Ministry of Science and Technology of China (no. 2019YFC1711100).

## Conflict of Interest

HY is employed by Yunnan Hemp Industrial Investment CO.LTD.

The remaining authors declare that the research was conducted in the absence of any commercial or financial relationships that could be construed as a potential conflict of interest.

## Publisher’s Note

All claims expressed in this article are solely those of the authors and do not necessarily represent those of their affiliated organizations, or those of the publisher, the editors and the reviewers. Any product that may be evaluated in this article, or claim that may be made by its manufacturer, is not guaranteed or endorsed by the publisher.

## Supplementary Material

The Supplementary Material for this article can be found online at: https://www.frontiersin.org/articles/10.3389/fpls.2022.904163/full#supplementary-material

## Footnotes

^1^ www.r-project.org

^2^ http://www.kegg.jp/kegg/compound/

^3^ http://www.kegg.jp/kegg/pathway.html

## References

[ref1] Abrahamsen F. Reddy G. Abebe W. Gurung N. (2021). Effect of varying levels of hempseed meal supplementation on humoral and cell-mediated immune responses of goats. Animals 11:2764. doi: 10.3390/ani11102764, PMID: 34679786 PMC8532981

[ref2] Aiello G. Fasoli E. Boschin G. Lammi C. Zanoni C. Citterio A.. (2016). Proteomic characterization of hempseed (*Cannabis sativa* L.). J. Proteome 147, 187–196. doi: 10.1016/j.jprot.2016.05.033, PMID: 27265319

[ref3] Andre C. M. Hausman J. Guerriero G. (2016). *Cannabis sativa*: the plant of the thousand and one molecules. Front. Plant Sci. 7:19 doi: 10.3389/fpls.2016.00019, PMID: 26870049 PMC4740396

[ref4] Benzie I. F. Strain J. J. (1996). The ferric reducing ability of plasma (FRAP) as a measure of “antioxidant power”: the FRAP assay. Anal. Biochem. 239, 70–76. doi: 10.1006/abio.1996.0292, PMID: 8660627

[ref5] Cattaneo C. Givonetti A. Leoni V. Guerrieri N. Manfredi M. Giorgi A.. (2021). Biochemical aspects of seeds from *Cannabis sativa* L. plants grown in a mountain environment. Sci. Rep. 11:3927. doi: 10.1038/s41598-021-83290-1, PMID: 33594196 PMC7887209

[ref6] Chen T. He J. Zhang J. Li X. Zhang H. Hao J.. (2012). The isolation and identification of two compounds with predominant radical scavenging activity in hempseed (seed of *Cannabis sativa* L.). Food Chem. 134, 1030–1037. doi: 10.1016/j.foodchem.2012.03.009, PMID: 23107724

[ref7] Dawidowicz A. L. Olszowy-Tomczyk M. Typek R. (2021). CBG, CBD, Δ9-THC, CBN, CBGA, CBDA and Δ9-THCA as antioxidant agents and their intervention abilities in antioxidant action. Fitoterapia 152:104915. doi: 10.1016/j.fitote.2021.104915, PMID: 33964342

[ref8] Fraga C. G. Clowers B. H. Moore R. J. Zink E. M. (2010). Signature-discovery approach for sample matching of a nerve-agent precursor using liquid chromatography-mass spectrometry, XCMS, and chemometrics. Anal. Chem. 82, 4165–4173. doi: 10.1021/ac1003568, PMID: 20405949

[ref9] Frassinetti S. Moccia E. Caltavuturo L. Gabriele M. Longo V. Bellani L.. (2018). Nutraceutical potential of hemp (*Cannabis sativa* L.) seeds and sprouts. Food Chem. 262, 56–66. doi: 10.1016/j.foodchem.2018.04.078, PMID: 29751921

[ref10] Galasso I. Russo R. Mapelli S. Ponzoni E. Brambilla I. M. Battelli G.. (2016). Variability in seed traits in a collection of *Cannabis sativa* L. Genotypes. Frontiers in Plant Science 7. doi: 10.3389/fpls.2016.00688, PMID: 27242881 PMC4873519

[ref11] Gomez C. C. Bermejo L. L. Loria K. V. (2011). Importance of a balanced omega 6/omega 3 ratio for the maintenance of health: nutritional recommendations. Nutr. Hosp. 26, 323–329. doi: 10.1590/S0212-16112011000200013, PMID: 21666970

[ref12] Hostetler G. L. Ralston R. A. Schwartz S. J. (2017). Flavones: food sources, bioavailability, metabolism, and bioactivity. Adv. Nutr. 8, 423–435. doi: 10.3945/an.116.012948, PMID: 28507008 PMC5421117

[ref13] House J. D. Neufeld J. Leson G. (2010). Evaluating the quality of protein from hemp seed (*Cannabis sativa* L.) products through the use of the protein digestibility-corrected amino acid score method. J. Agric. Food Chem. 58, 11801–11807. doi: 10.1021/jf102636b, PMID: 20977230

[ref14] Hurgobin B. Tamiru-Oli M. Welling M. T. Doblin M. S. Bacic A. Whelan J.. (2021). Recent advances in *Cannabis sativa* genomics research. New Phytol. 230, 73–89. doi: 10.1111/nph.17140, PMID: 33283274 PMC7986631

[ref01] Irakli M. Tsaliki E. Kalivas A. Kleisiaris F. Sarrou E. Cook C. M. (2019). Effect of genotype and growing Yearon the nutritional, phytochemical, and antioxidant properties of industrial hemp (*Cannabis sativa* L.) seeds. Antioxidants 8:491. doi: 10.3390/antiox8100491 PMC6826498 31627349

[ref15] Kovalchuk I. Pellino M. Rigault P. van Velzen R. Ebersbach J. Ashnest J. R.. (2020). The genomics of cannabis and its close relatives. Annu. Rev. Plant Biol. 71, 713–739. doi: 10.1146/annurev-arplant-081519-040203 32155342

[ref16] Kumari S. (2022). Investigating the antioxidant and anticancer effect of alkaloids isolated from root extracts of *Berberis aristata*. Chemical Data Collections 37:100805. doi: 10.1016/j.cdc.2021.100805

[ref17] Li W. Wen L. Chen Z. Zhang Z. Pang X. Deng Z.. (2021). Study on metabolic variation in whole grains of four proso millet varieties reveals metabolites important for antioxidant properties and quality traits. Food Chem. 357:129791. doi: 10.1016/j.foodchem.2021.129791, PMID: 33895687

[ref18] Madunic J. Madunic I. V. Gajski G. Popic J. Garaj-Vrhovac V. (2018). Apigenin: a dietary flavonoid with diverse anticancer properties. Cancer Lett. 413, 11–22. doi: 10.1016/j.canlet.2017.10.041, PMID: 29097249

[ref19] Majewski M. Jurgoński A. (2021). The effect of hemp (*Cannabis sativa* L.) seeds and hemp seed oil on vascular dysfunction in obese male zucker rats. Nutrients 13:2575. doi: 10.3390/nu13082575, PMID: 34444734 PMC8398088

[ref20] Park S. Seo J. Lee M. (2012). Proteomic profiling of hempseed proteins from Cheungsam. Biochimica Et Biophysica Acta (BBA) - Proteins and Proteomics 1824, 374–382. doi: 10.1016/j.bbapap.2011.10.005, PMID: 22040604

[ref21] Piazzon A. Vrhovsek U. Masuero D. Mattivi F. Mandoj F. Nardini M. (2012). Antioxidant activity of phenolic acids and their metabolites: synthesis and antioxidant properties of the sulfate derivatives of ferulic and caffeic acids and of the acyl glucuronide of ferulic acid. J. Agric. Food Chem. 60, 12312–12323. doi: 10.1021/jf304076z, PMID: 23157164

[ref22] Rashid A. Ali V. Khajuria M. Faiz S. Gairola S. Vyas D. (2021). GC–MS based metabolomic approach to understand nutraceutical potential of Cannabis seeds from two different environments. Food Chem. 339:128076. doi: 10.1016/j.foodchem.2020.128076, PMID: 33152869

[ref23] Ren G. Zhang X. Li Y. Ridout K. Serrano-Serrano M. L. Yang Y.. (2021). Large-scale whole-genome resequencing unravels the domestication history of *Cannabis sativa*. Sci. Adv. 7:eabg2286. doi: 10.1126/sciadv.abg2286, PMID: 34272249 PMC8284894

[ref24] Schluttenhofer C. Yuan L. (2017). Challenges towards revitalizing hemp: a multifaceted crop. Trends Plant Sci. 22, 917–929. doi: 10.1016/j.tplants.2017.08.004, PMID: 28886910

[ref25] Simopoulos A. P. (2008). The importance of the omega-6/omega-3 fatty acid ratio in cardiovascular disease and other chronic diseases. Exp. Biol. Med. (Maywood) 233, 674–688. doi: 10.3181/0711-MR-311, PMID: 18408140

[ref26] Tena N. Martin J. Asuero A. G. (2020). State of the art of anthocyanins: antioxidant activity, sources, bioavailability, and therapeutic effect in human health. Antioxidants 9:451. doi: 10.3390/antiox9050451, PMID: 32456252 PMC7278599

[ref27] Xie B. Shi H. Chen Q. Ho C. T. (1993). Antioxidant properties of fractions and polyphenol constituents from green, oolong and black teas. Proc. Natl. Sci. Counc. Repub. China B 17, 77–84. 7809277

[ref28] Yonekura-Sakakibara K. Yamamura M. Matsuda F. Ono E. Nakabayashi R. Sugawara S.. (2021). Seed-coat protective neolignans are produced by the dirigent protein AtDP1 and the laccase AtLAC5 in Arabidopsis. Plant Cell 33, 129–152. doi: 10.1093/plcell/koaa014, PMID: 33751095 PMC8136895

[ref29] Zhang B. Horvath S. (2005). A general framework for weighted gene co-meta network analysis. Stat. Appl. Genet. Mol. Biol. 4:e17. doi: 10.2202/1544-6115.1128 16646834

[ref30] Zhang Z. Shi Q. Wang B. Ma A. Wang Y. Xue Q.. (2021). Jujube metabolome selection determined the edible properties acquired during domestication. Plant J. 109, 1116–1133. doi: 10.1111/tpj.15617, PMID: 34862996

