# Supplementary material for: Metabolomics Analysis Revealed the Characteristic Metabolites of Hemp Seeds Varieties and Metabolites Responsible for Antioxidant Properties
Source: Front Plant Sci. 2022 Jun 21;13:904163. doi: 10.3389/fpls.2022.904163 (PMC9253560; doi:10.3389/fpls.2022.904163)
Supplement: Supplementary file 1 [file Table_1.DOCX]

Supplementary Material

Comprehensive analysis of metabolites in different hempseed varieties

**Kang Ning^1#^, Cong Hou^1#^, Xiuye Wei^1^, Yuxin Zhou^1^, Shuanghua Zhang^1^, Yongzhong Chen^1^, Haibin Yu^2^, Linlin Dong^1*^, Shilin Chen^1**^.**

*1. Key Laboratory of Beijing for Identification and Safety Evaluation of Chinese Medicine, Institute of Chinese Materia Medica, China Academy of Chinese Medical Sciences; Beijing 100700, China;*

*2. Yunnan Hemp Industrial Investment CO.LTD; Kunming 650217, China.*

# These authors have contributed equally to this work and share first authorship

## Supplementary Figures


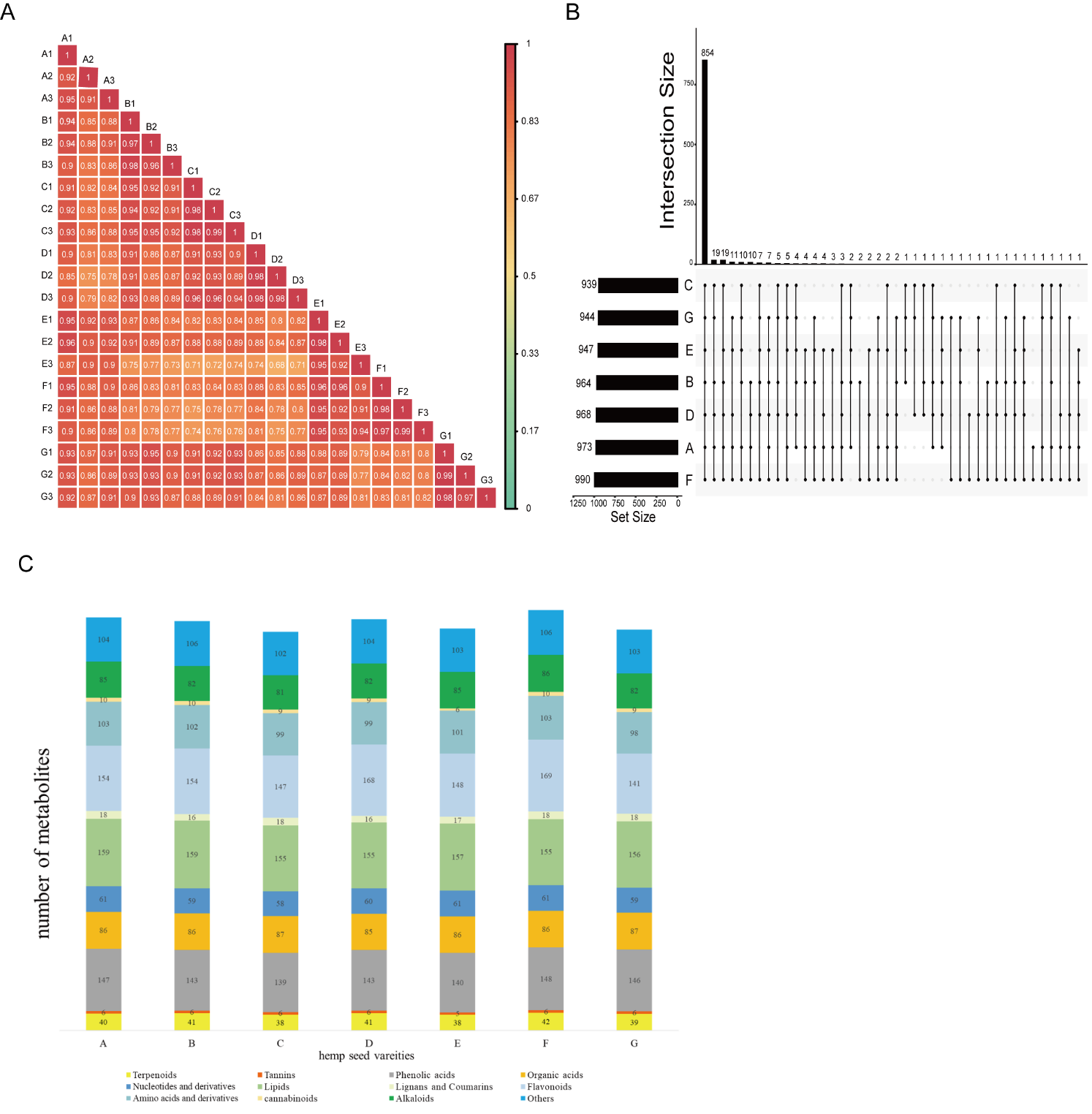


Fig. S1 Overview of the detected metabolites in the seven hempseed varieties.

**A**. Correlation of each sample. **B**. Venn diagram of metabolite distribution in different varieties. **C**. Distribution of metabolites in different varieties.

Supplementary Table 1: Details of the locations of the seven hemp seed varieties.

Supplementary Table 2: Metabolites identified in the metabolome.

Supplementary Table 3: Comparison of different varieties in detail.
